# Great auricular neuralgia: case series

**DOI:** 10.1186/1129-2377-14-S1-P161

**Published:** 2013-02-21

**Authors:** CE Robertson, I Garza

**Affiliations:** 1 Mayo Clinic, Rochester, MN, USA

## Introduction

The great auricular nerve (GAN) leaves the C2-C3 cervical rami, wraps around the sternocleidomastoid muscle, then divides into: anterior branch (skin over preauricular, parotid, jaw angle) and posterior branch (skin over mastoid and posteroinferior pinna). GAN damage is well described with procedures nearby the nerve course. However, neuralgia of this nerve is uncommon, and its knowledge is based on a handful of case reports.

## Objective

Describe the presentation, treatment, and outcome of 6 great auricular neuralgia cases.

## Methods

We reviewed charts from 1994-2010 with diagnoses: auricular neuralgia, neuritis, or neuropathy. We included subjects with neuralgia restricted to GAN distribution, without associated neuropathy. Cases with missing information or pain in alternative nerve distributions were excluded.

## Results

Of 59 charts, 6 patients met criteria (ages 11-59, all female). Presumed etiologies: low-grade lymphoma of GAN, Sjögren’s syndrome, trauma from tympanomastoid procedures (n=2), and idiopathic (n=2). All patients had paroxysmal shock-like pain in the territory of the GAN. Three also had persistent pain, described as burning (n=2) or dull (n=1). Pain provoked by: turning the head ipsilaterally (n=3), neck position during sleep (n=3), exertion (n=1), lifting with ipsilateral arm (n=1).

Treatments varied, including various neuropathic medicines without relief. The lymphoma patient had resolution of pain following nerve resection. Three patients received GAN blocks: all noted dramatic improvement in their pain. One was successfully treated with serial blocks over 2.5 years. The other two transitioned from GAN blocks to GAN stimulators with almost complete resolution of their pain.

## Conclusion

Great auricular neuralgia should be considered in the differential for paroxysmal stabbing periauricular pain. Like other craniocervical neuralgias, it may be idiopathic or secondary to underlying pathology. More study is needed, but it may be reasonable to consider GAN blocks or stimulators in treatment of these cases.
